# Nullity of GSTM1 and GSTT1 Associated with CD4+ T Cells in HIV-Positive Patients from Southern Brazil

**DOI:** 10.3390/antiox14080909

**Published:** 2025-07-25

**Authors:** Marcela Gonçalves Trevisan, Marcieli Borba do Nascimento, Valdir Spada Juníor, Volmir Pitt Benedetti, Lirane Elize Defante Ferreto, Léia Carolina Lucio

**Affiliations:** 1 Postgraduate Program in Applied Health Sciences, State University of West Paraná, Unioeste, Francisco Beltrão 85.601-839, Brazil; marcelag.trevisan@gmail.com (M.G.T.); marcieli_bn@hotmail.com (M.B.d.N.); lirane.ferreto@unioeste.br (L.E.D.F.); 2 Undergraduate Course in Medicine, State University of West Paraná, Unioeste, Francisco Beltrão 85.601-839, Brazil; valdir.junior3@unioeste.br; 3 Paraná University, Unipar, Francisco Beltrão 85.601-000, Brazil; volmir@unipar.br; 4 Laboratory of Molecular Biology and Human Genetics, State University of West Paraná, Unioeste, Francisco Beltrão 85.601-839, Brazil

**Keywords:** GSTM1, GSTT1, CD4+ T lymphocyte, glutathione S-transferase, HIV

## Abstract

Scientific evidence has suggested, in most cases, that nullity of the GSTM1 and GSTT1 genes is associated with worse pathological outcomes and viral infections. In this sense, the main objective of this work was to determine the genotypic frequencies of GSTM1 and GSTT1 polymorphisms in individuals with HIV and to establish a possible relationship with CD4+ T lymphocyte count. This was a cross-sectional study, with a quantitative approach, composed of 182 HIV-positive patients. To detect GSTM1 and GSTT1 polymorphisms by the multiplex polymerase chain reaction (PCR), oral mucosa samples were collected. Regarding genotypic frequencies, GST nullity was high in the population, being 97.5% and 97.6%, respectively, for GSTM1− and GSTT1−. Although there was no association between the GST polymorphism and the viral load and CD4+ T lymphocyte counts at diagnosis, when related to the current CD4+ count, the isolated and combined null alleles, GSTT1 (ORadj: 0.219; *p* = 0.004), GSTM1 (ORadj: 0.219; *p* = 0.004), and GSTM1/T1 (ORadj: 0.219; *p* = 0.004), were defined as factors favorable to a minimum CD4+ T lymphocyte count of 350 cells. Therefore, this study demonstrated a probable relationship between the GSTT1 and GSTM1 genetic polymorphisms and HIV.

## 1. Introduction

The human immunodeficiency retrovirus (HIV) is capable of attacking the immune system of individuals, weakening it and causing acquired immune deficiency syndrome (AIDS), which leads to the emergence of opportunistic diseases [1]. Since their discovery, HIV and AIDS have become one of the leading causes of death worldwide. The first cases of the syndrome were discovered and recorded in the United States of America (USA), Haiti, and Central Africa around the 1970s. And, in Brazil, the first record of HIV infection was in the 1980s, in the city of São Paulo [2]. Between 2007 and mid-2021, 381,793 cases of HIV infection were reported in the Notifiable Diseases Information System (SINAN), of which 75,165 (19.7%) were in the southern region. Furthermore, according to the HIV/AIDS Epidemiological Bulletin released at the end of 2021, the majority of HIV infections affected the 20 to 34 age group (52.9%) [3].

The incidence of HIV-related deaths and the development of AIDS are due to a continuous immunodeficiency associated with the depletion of CD4+ T lymphocytes, compromising the immune system [4]. As this is a disease with a high fatality rate, early diagnosis and effective treatment are extremely important to minimize complications resulting from the infection. However, despite the various advances involving actions in the prevention, diagnosis, and treatment of AIDS, HIV infection remains a global challenge, as it has undergone an intense epidemiological transition from an acute disease to a chronic condition [5].

In turn, glutathione-S-transferases (GSTs) are a family of enzymes that act in phase II of xenobiotic metabolism and play a significant role in gene–environment interactions, cell signaling, anti-apoptotic activity, and anti- and pro-inflammatory responses. In total, GSTs comprise at least eight classes of isoenzymes: alpha (A), kappa (K), mu (M), omega (O), pi (P), sigma (S), theta (T), and zeta (Z) [6]. Genetic polymorphisms in this enzyme group can interfere with the metabolism of various environmental agents, increase oxidative stress and susceptibility to pathologies, and favor viral replication, including HIV [7,8,9,10,11]. Among the genes, GSTM1 and GSTT1 are the most studied in the human population [12]. GSTM1 is located on chromosome 1p13.3 and GSTT1 on 22q11.23 [13,14] and polymorphic variations characterize the expression of wild-type alleles or the absence of expression when the alleles are null/deleted [12,15,16].

In general terms, GST gene variants alter the catalytic function of enzymes and increase the risk of adverse outcomes from a given disease [17]. However, there is no consensus on this relationship. A study conducted in Ghana and India found frequencies of GSTM1 and GSTT1 null alleles of 21.9% and 19.8%, respectively, in patients with HIV and observed that those with double deletion were associated with normal CD4+ T lymphocyte counts (above 350 cells/mm^3^) [18]. Research [19] demonstrated that GSTT1 deletion was more frequent in individuals with HIV compared with those not infected. The authors also suggested that the GSTT1/GSTM1 double deletion may predict a higher risk of hepatotoxicity in individuals with the virus. In contrast, another study showed a lack of significance in the GSTT1 and GSTM1 genotypic frequencies among people living with or not living with HIV. However, it was observed that patients with HIV and the GSTT1+ allele were twice as likely to develop liver injury induced by antiretroviral therapy (ART) [20]. Another controversial study on the relationship between gene nullity and unfavorable outcomes of viral infections concluded that the GSTT1 null genotype may be a protective factor against HPV infection in a group of women [21]. Given the above, the main objective of this study was to determine the genotypic frequencies of the GSTM1 and GSTT1 polymorphisms in individuals with HIV and to establish a possible relationship with CD4+ T lymphocyte counts.

## 2. Materials and Methods

### 2.1. Study Population

The sampling was conducted by convenience, consisting of 182 HIV-positive patients who attended the Specialized Care Service and Testing and Counseling Center (SCS/TCC) in the municipality of Francisco Beltrão, which serves the 8th Health Region of the state of Paraná, between 2020 and 2022. Individuals aged at least 18 years and HIV/AIDS carriers who agreed to the study and signed the Informed Consent Form (ICF) were included in the study. This research was approved by the Ethics Committee for Research Involving Human Beings of the State University of Western Paraná under opinion number 3,611,523. The participants were allocated to a restricted space to conduct the interview using a questionnaire constructed from others that had already been validated [22,23] and the collection of biological material. The biological sample for the investigation of GST polymorphisms was obtained from cells of the patients’ oral mucosa. This collection was performed with a sterile endobrush, using delicate circular movements across the entire length of the oral mucosa and tongue. The brush was then stored in a 15 mL tube containing up to 2 mL of buffer solution (Tris-HCL 10 mM, pH 8, and EDTA 1 mM), duly identified, and stored at −20 °C for later processing [24]. It should be noted that the initial sample consisted of 279 patients; however, 97 were excluded due to the low quality of the oral material collected and the absence of genetic material or because they did not adequately answer all the questions in the questionnaire.

### 2.2. Isolation of Genetic Material or Extraction of Genetic Material/DNA

Total DNA extraction from each individual was performed from a 200 µL aliquot of the buffer solution in which the brush was stored following the QIAamp^®^ DNA Mini Kit, DNA Purification from Blood or Body Fluids (Spin Protocol) (Qiagen^®^, Hilden, Germany), according to the manufacturer’s instructions and kept in a −20 °C freezer. After DNA extraction, the presence of a 268 bp segment of the human β-globin gene (Gene ID: 3043) synthesized from primers GH20 (5′-GAAGAGCCAAGGACAGGTAC-3′) and PC04 (5′- CAACTTCATCCACGTTCACC-3′) was detected to verify the presence and quality of the DNA in the samples. The PCR reagents were 190 nM dNTPs, 500 nM of each primer, 2 mM MgCl2, buffer (200 mM Tris-HCL, 500 mM KCl), 1.25 U of DNA polymerase (Ludwig Biotec^TM^, Alvorada, Brazil), and approximately 50 ng of DNA subjected to the following cycling stages: 94 °C for 10 min; 37 cycles of 94 °C for 1 min, 55 °C for 1 min, 72 °C for 1 min; and 72 °C for 10 min [25]. Subsequently, the amplicons were observed via electrophoresis in a 2% agarose gel and stained with ethidium bromide.

### 2.3. GSTM1 (Gene ID: 2944) and GSTT1 (Gene ID: 2952) Genotyping

GSTM1 and GSTT1 genotypes were determined via multiplex PCR [21]. The primer pairs used were 5′-GAACTCCCTGAAAAGCTAAAGC-3′ (forward) and 5′-GTTGGGCTCAAATA TACGGTGG-3′ (reverse) for GSTM1 and 5′-TTCCTTACTGGTCCTCACATCTC-3′ (forward) and 5′-TCACCGGATCATGGCCAG CA-3′ (reverse) for GSTT1, with respective synthesis of 219 bp and 459 bp fragments. The multiplex PCR conditions included initial denaturation at 94 °C for 5 min, followed by 35 cycles at 94 °C for 1 min, 58 °C for 1 min, 72 °C for 1 min, and ending with 10 min at 72 °C. The synthesized GST amplicons were also electrophoresed under similar conditions as the β-globin gene. All DNA amplifications were processed in an Applied Biosystems Veriti Thermal Cycler (ThermoFisher Science, Karlsruhe, Germany).

### 2.4. Statistical Analysis

In the descriptive analysis, the mean and standard deviation were established for continuous variables and the absolute and relative frequency for categorical variables. The GSTM1 and GSTT1 genotype frequency and the association of genotypes with CD4+ T lymphocyte count at diagnosis and current were assessed by Pearson’s Chi-square test (X^2^), considering *p* < 0.20. Subsequently, logistic regression was conducted with 95% significance and a 95% confidence interval (CI). All analyses were processed in the Statistical Package for the Social Sciences—SPSS software, version 24.0 (IBM Corporation, Chicago, IL, USA).

## 3. Results

A total of 182 HIV-positive patients participated in this study. Among the general aspects of the HIV population, there was a predominance of female individuals (56.6%), white (57.1%), heterosexual (80.8%), married (38.5%), and with low education (47.3%), and the mean age was 43.98 ± 12.8 years.

The data presented in Table 1 indicate the presence of sexually active patients (74.7%) who practiced unprotected sexual relations, as 33.5% did not use condoms and 18.7% use the method sporadically, with low adherence being justified by having a steady partner (19.2%) or by not liking the method of protection (32.4%). Furthermore, it was identified that less than 24% of the patients had been diagnosed with AIDS. Regarding transmission, the majority occurred through sexual contact (98.4%), with partners being aware of the diagnosis (75.3%) and being HIV-positive (42.9%).

Among the antiretroviral regimens most commonly prescribed, the combinations of Tenofovir, Lamivudine, and Dolutegravir (46.2%) and Tenofovir, Lamivudine, and Efavirenz (16.5%) stood out.

An analysis of the genotypic frequencies of GSTM1 and GSTT1 revealed null alleles for most study participants. Considering the null alleles, they had a higher prevalence compared with the wild-type alleles, at 95.1% and 91.2%, respectively, for GSTT1^−^ and GSTM1^−^. Consequently, among the GST combinations, a predominance of the double-deleted genotype M1^−^/T1^−^ (89.6%) was identified (Table 2).

The relationship between GST genotypes and some sexual behaviors of HIV patients did not reveal a statistical association. However, among individuals with a genotypic combination of deletions (GSTT1^−^ and GSTM1^−^), the majority started sexual activity before the age of 18 (73.3%), had more than five sexual partners throughout their lives (50.3%), and even had a new partner in the last year (85.7%).

The association between GST genotypes and the viral load of patients also did not reveal significant data. However, it is worth noting that the majority of patients who presented a viral load equal to or greater than 500 copies/mL at diagnosis were those with the double deletion GSTT1 and GSTM1 (62.3%). In addition, the genotypic combination M1^−^/T1^−^ predominated among those with an undetectable viral load (48.1%) in the last test, followed by those who presented a viral load below 500 copies/mL (44.4%).

Regarding the association of GST genotypes with CD4+ T cell counts, Table 3 and Table 4 present the results of the regression analysis. No association was identified between the GST polymorphism and CD4+ T cell counts at diagnosis. On the other hand, when related to the current count, individuals with the null alleles GSTT1, GSTM1, and the combination M1^−^/T1^−^ had a greater chance of having minimum CD4+ T cell counts, equivalent to 350 cells/mm^3^, when compared with wild-type alleles. For the GSTT1^−^ genotype, the chance was increased by more than 12 times (OR 12.33; *p* = 0.001). And for GSTM1^−^ and the double deletion M1^−^/T1^−^, the odds were almost four times (OR: 3.53; *p* = 0.025) and ten times (OR: 10.07; *p* = 0.014). These findings, therefore, suggest that GST nullity in HIV patients makes them less susceptible to progression or development of AIDS.

## 4. Discussion

Molecular analysis of GSTM1 and GSTT1 genotype frequencies demonstrated that most study participants had allelic nullity. Consequently, among the GST combinations, a predominance of double-deleted genotypes (M1^−^/T1^−^) was identified. It is emphasized that comparisons between the frequencies of GSTM1 and GSTT1 null genotypes in world populations show patterns different from this study’s findings. According to a literature review, it is estimated that the worldwide frequencies of GSTT1 and GSTM1 deletion range from 10% to 51% and from 11% to 67%, respectively [26].

In turn, a study carried out in the same geographic region, southwest of Paraná, southern Brazil, revealed, in both women infected and uninfected by the STI caused by the human papillomavirus (HPV), a higher frequency of the GSTM1 null allele in both groups, at 57.1% and 75%. On the other hand, the GSTT1 null allele was less frequent in the HPV group (38.1%) compared with the control (73.8%) [21].

Conflicting data were found in an American study [27], when only 41.2% of HIV individuals had null GSTM1, and in an African study [28], since the proportions of GSTM1 and GSTT1 null alleles in the HIV population were 30.35% and 35.46%, respectively. However, similarly, it was found that individuals with GSTM1 and GSTT1 null genotypes were more present in the HIV-1-positive group and the difference between the two groups was significant, respectively, at 37.25% versus 23.75% for GSTM1 null and 48.37% versus 23.10% for GSTT1 null. Furthermore, a significant difference was also found between cases and controls in relation to the GSTM1^−^/GSTT1^−^ double deletion genotype.

In addition, research conducted with the healthy population of Mali, Africa, also identified not very significant frequencies of the GSTM1 and GSTT1 deletions of only 24.3% and 41.3%, and found that the mean frequency of the GSTM1 null allele increased from the South to the East in the country, while that of the GSTT1 deletion was similar between the West and East and South and North [29].

Considering that allelic variations among individuals are very frequent, the use of genetic markers has been gaining greater credibility, acceptance, and application in population studies including various pathologies [30]. Single-nucleotide polymorphisms (SNPs) are characterized by a single exchange of a nitrogenous base in DNA, generally with the same structural characteristics, that is, between purines or between pirimidines [31], and represent 90% of all genomic variations in humans. In fact, to be considered, they must occur in at least 1% of the population [32].

The SNPs targeted by our study, GSTM1 and GSTT1, are susceptible to deletion and complete loss of enzymatic activity and are related to coronary diseases [33], different types of cancer [8,34], and various infections [9,15,27,35]. They also alter the detoxification metabolism of many substrates, such as anticancer and anti-infectious drugs. Therefore, studies that seek to characterize these genotypes may reveal differences in genetic and pharmacogenetic susceptibility between populations [29].

Among the antiretroviral regimens preferentially prescribed to the research participants, the combinations involving Tenofovir, Lamivudine, and Dolutegravir and Tenofovir, Lamivudine, and Efavirenz stand out. According to the manual for adherence to treatment for people living with HIV and AIDS from the Ministry of Health, HIV infection has been considered chronic, progressive, and potentially controllable since the emergence of ART and the availability of biological markers, such as CD4+ T cells and quantification of viral load, to monitor its progression [36]. By reducing viral load and reconstructing the immune system, treatment improves quality of life and prolongs the survival of individuals with the virus. Several studies have also demonstrated the efficacy of ART, mainly by reducing mortality and complications related to HIV infection, in addition to preventing its transmission [37,38,39].

A study that sought to describe the impact of genetic polymorphisms of GSTM1 and GSTT1 and the risk of hepatotoxicity associated with ART indicated a predominance of the GSTT1− genotype frequency in HIV-positive individuals when compared with uninfected individuals, and that the double deletion of GSTT1 and GSTM1 may confer a greater risk of hepatotoxicity in the HIV population [19]. A study carried out with HIV-positive individuals in Mozambique, treated with nevirapine, identified that the development of Stevens–Johnson syndrome and toxic epidermal necrolysis was associated with the GSTM1 null allele [40]. Whereas, research conducted in the Argentine capital, which evaluated the role of the GSTT1 and GSTM1 variants with porphyria cutanea tarda (PCT) suggested that the presence of the GSTM1 wild allele could predispose the individual with HIV to the development of the disease, since the GSTM1 null genotype presented a significantly lower frequency in the group of HIV patients with PCT (36.84%) when compared with the group that only contained HIV (53.33%) [41].

In contrast, another study, although it did not indicate an association between the GSTT1 and GSTM1 genotype frequencies and people living with or not living with HIV, showed that the presence of the wild-type GSTT1 allele doubled the chances of HIV patients developing ART-induced liver damage [20]. It is known that prolonged HIV infection contributes to the exacerbation of oxidative stress [42]. Thus, the presence of the active form of the GSTT1 gene would, as a rule, contribute to cellular defense; however, this allelic form in some populations can act in reverse, accentuating the damage [20].

The relationship between GST genotypes and current CD4+ T cell counts showed that isolated or combined GST allele nullity is a protective factor in this population. It is known that HIV is responsible for causing a progressive deterioration of the immune system, infecting mainly CD4+ T lymphocytes, macrophages, and dendritic cells [36]. AIDS, in turn, is the result of a progressive drop in the CD4+ TL count, resulting from a severe state of immunodeficiency, especially when the number of CD4+ TL falls below 200 cells/mm^3^, weakening the individual and favoring opportunistic infections and even the development of neoplasias [4].

In view of this, the findings of the study allow us to infer that patients with a null genotypic profile are less susceptible to AIDS, since allelic deletions increase the chances of CD4+ TL counts being greater than or equal to 350 cells/mm^3^. Furthermore, considering that the CD4+ T lymphocyte counts remain above 350 cells/mm^3^, the occurrence of infectious episodes, usually of bacterial origin, such as respiratory infections or even tuberculosis [43], is also reduced, possibly justifying the low prevalence of opportunistic infections in the population.

Similar data were observed in HIV-positive patients in Ghana, since HIV carriers who had both alleles deleted had normal CD4+ T lymphocyte counts, that is, above 350 cells/mm^3^ [18]. In fact, a case–control study conducted by researchers involved in the present study found a favorable association of the GSTT1− genotype with a reduced risk of HPV infection in a group of women [21].

In contrast, research that investigated the association between GSTM1 and GSTT1 gene polymorphisms and the risk of HIV-1 disease progression in Africa found a statistically significant difference when comparing GSTM1 and GSTT1 frequencies with CD4+ T lymphocyte counts and viral load, suggesting that both null genotypes and the GSTM1^−^/GSTT1^−^ double deletion are associated with disease progression. It is also worth noting that the GSTM1 null genotype and the GSTM1^−^/GSTT1^−^ double deletion were associated with low CD4+ T cell counts and high HIV-1 viral load in infected patients using ART [28]. Another contradictory result to ours revealed that immunosuppressed patients in Florida, USA, with the GSTM1 wild-type allele had less severity and progression of the disease caused by HIV [27]. These data undoubtedly do not match the findings of the present study.

This divergence in findings can be attributed to several factors. First, it is important to consider that the distribution of genetic polymorphisms varies significantly among different population groups, which may influence the functional effects of these variants. In addition, environmental and behavioral factors, such as the presence of co-infections, use of psychoactive substances, and adherence to antiretroviral treatment, may also act as confounding variables, modulating the clinical expression of polymorphisms.

In this context, the lack of association observed in our study may reflect the complex interaction between genetic, clinical, and environmental factors. These aspects highlight the importance of multicenter studies, with larger samples and rigorous control of confounding variables, for a more comprehensive understanding of the role of the GSTM1 and GSTT1 genes in the progression of HIV infection.

The main limitation of this study is the sample size, which gives it low power of generalization, and the cross-sectional design, which, although it met the objective of this study, does not allow for monitoring the manifestations developed by the patients.

## 5. Conclusions

In our study, it was possible to establish the influence of the nullity of the GSTT1 and GSTM1 genotypes on HIV infection. The results were unprecedented in the region and demonstrated that the presence of the GSTT1 and GSTM1 null alleles, isolated and combined, in this population was a protective factor, increasing the chances of CD4+ T lymphocyte counts equal to or greater than 350 cells/mm^3^. Thus, it is suggested that HIV patients with GSTT1 and GSTM1 deletions are less susceptible to the progression of AIDS. On the other hand, as there is no consensus on the relationship between nullity and outcomes related to HIV infection, this interpretation is restricted to the study population. Considering the limitations of this research, we emphasize the need to continue the work on a longitudinal scale and to structure a case–control study.

## Figures and Tables

**Table 1:** Clinical, behavioral, and lifestyle characteristics of HIV-positive patients treated at the Specialized Care Service of Southwest Paraná, Francisco Beltrão, PR, Brazil, 2022.

Variables	Participants (n = 182)	Valid Frequency (%)
Age of sexarche		
<18 years	126	69.2
≥18 years	56	30.8
Active sex life		
Yes	136	74.7
No	46	25.3
Condom use		
Yes	87	47.8
No	61	33.5
Sometimes	34	18.7
Why does not use a condom		
I do not like	59	32.4
Steady partner	35	19.2
Not applicable	88	48.4
Sexual partners in life		
≤5 partners	95	52.2
>5 partners	87	47.8
New sexual partners in the last year		
≤1 partner	156	85.7
≥2 partners	26	14.3
STI History		
Yes	64	35.2
No	118	64.8
Opportunistic infections		
Yes	30	16.5
No	152	83.5
Chronic diseases		
Yes	92	50.5
No	90	49.5
Smoking		
Yes	86	47.3
No	96	52.7
Number of cigarettes/day		
Up to 20 cigarettes/day	67	36.8
≤21 cigarettes/day	19	10.4
Not applicable	96	52.7
Age when started smoking		
Up to 20 years	73	40.1
≤21 years	13	7.1
Not applicable	96	52.7
Alcohol intake in the last year		
Yes	107	58.8
No	75	41.2
Use of illicit drugs at the time of infection		
Yes	23	12.6
No	159	87.4
Current use of illicit drugs		
Yes	8	4.4
No	174	95.4
Blood transfusion		
Yes	36	19.8
No	146	80.2
Way you acquired HIV		
Sexual	179	98.4
Others	3	1.6
Aware partner		
Yes	137	75.3
No	39	21.4
Does not know	6	3.3
Partner serology		
Positive	78	42.9
Negative	52	28.6
Undetermined	52	28.6
Morbidity at diagnosis		
HIV	140	76.9
AIDS	42	23.1
Perception of health status		
Excellent/Very good	36	19.8
Good	91	50.0
Regular/poor	55	30.2

STI: sexually transmitted infection. HIV: human immunodeficiency virus.

**Table 2:** GST genotypic frequency in HIV-positive patients in this study.

Genotypes	Participants (N = 182)	Frequency (%)
GSTM1		
M1^+^	16	8.8
M1^−^	166	91.2
GSTT1		
T1^+^	9	4.9
T1^−^	173	95.1
GST Combinations		
M1^+^/T1^+^	5	2.7
M1^−^/T1^+^	11	6.1
M1^+^/T1^−^	3	1.6
M1^−^/T1^−^	163	89.6

GST: glutathione-S-transferases. M1^+^ and T1^+^: allele present. M1^−^ and T1^−^: allele absent.

**Table 3:** Absence of association of GST genotypes with CD4+ T cell count (cell/mm^3^) in the diagnosis of HIV virus.

Polymorphism GST	CD4+ T Cell Count at Diagnosis			Odds Ratio	CI 95%	*p*
	<350 cell/mm^3^	≥350 cell/mm^3^	*p*			
	N (%)	N (%)				
GSTT1^+^	4 (44.4)	5 (55.6)	0.733	1		
GSTT1^−^	65 (37.8)	107 (62.2)		1.317	(0.34–5.08)	0.689
GSTM1^+^	5 (31.2)	11 (68.8)	0.603	1		
GSTM1^−^	64 (38.8)	101 (61.2)		0.717	(0.24–2.16)	0.555
*Combinations*						
GSTT1^+^/GSTM1^+^	1 (20.0)	4 (80.0)	0.614	1		
GSTT1^−^/GSTM1^+^	5 (45.5)	6 (54.5)		0.3	(0.03–3.63)	0.344
GSTT1^+^/GSTM1^−^	2 (66.7)	1 (33.3)		0.125	(0.005–3.23)	0.210
GSTT1^−^/GSTM1^−^	61 (37.7)	101 (62.3)		0.414	(0.045–3.79)	0.435

GST: glutathione-S-transferases. M1^+^ and T1^+^: allele present. M1^−^ and T1^−^: allele absent. CI: confidence interval.

**Table 4:** Association of GST genotypes with current CD4+ cell count equal to or greater than 350 cells/mm^3^.

Polymorphism GST	Current CD4+ T Cell Count			Odds Ratio	CI 95%	*p*
	<350 cell/mm^3^	≥350 cell/mm^3^	*p*			
	N (%)	N (%)				
GSTT1^+^	6 (66.7)	3 (33.7)	0.001	1		
GSTT1^−^	24 (14.0)	148 (86.0)		12.33	(2.89–52.65)	0.001
GSTM1^+^	6 (37.5)	10 (62.5)	0.030	1		
GSTM1^−^	24 (14.5)	141 (85.5)		3.53	(1.17–10.60)	0.025
*Combinations*						
GSTT1^+^/GSTM1^+^	3 (60.0)	2 (40.0)	0.001	1		
GSTT1^−^/GSTM1^+^	4 (36.4)	7 (63.6)		2.63	(0.3–22.99)	0.383
GSTT1^+^/GSTM1^−^	2 (66.7)	1 (33.3)		0.75	(0.38–14.97)	0.851
GSTT1^−^/GSTM1^−^	21 (13.0)	141 (87.0)		10.07	(1.59–63.86)	0.014

GST: glutathione-S-transferases. M1+ and T1+: allele present. M1^−^ and T1^−^: allele absent. CI: confidence interval.

## Data Availability

Dataset available on request from the authors.

## Abbreviations

The following abbreviations are used in this manuscript:

AIDS	Acquired immunodeficiency syndrome
ART	Antiretroviral therapy
GST	Glutathione-S-transferases
HIV	Human immunodeficiency virus
ICF	Informed consent form
PCR	Polymerase chain reaction
PCT	Porphyria cutanea tarda
SCS/TCC	Specialized Care Service and Testing and Counseling Center
SINAN	Notifiable Diseases Information System
SNP	Single-nucleotide polymorphisms
USA	United States of America
